# Association Between Childhood Neighborhood Quality and the Risk of Cognitive Dysfunction in Chinese Middle-Aged and Elderly Population: The Moderation Effect of Body Mass Index

**DOI:** 10.3389/fnagi.2021.645189

**Published:** 2021-05-13

**Authors:** Peng Xiong, Xiaohua Liang, Haiyan Chen, Li Chen, Lei Zuo, Chunxia Jing, Guang Hao

**Affiliations:** ^1^Division of Medical Psychology and Behavioral Sciences, Department of Public Health and Preventive Medicine, School of Medicine, Jinan University, Guangzhou, China; ^2^Clinical Epidemiology and Biostatistics Department, Children’s Hospital of Chongqing Medical University, Ministry of Education Key Laboratory of Child Development and Disorders, National Clinical Research Center for Child Health and Disorders, Key Laboratory of Pediatrics in Chongqing, International Science and Technology Cooperation Center of Child Development and Critical Disorders, Chongqing, China; ^3^Guangzhou Center for Disease Control and Prevention, Guangzhou, China; ^4^Georgia Prevention Institute, Department of Population Health Sciences, Medical College of Georgia, Augusta University, Augusta, GA, United States; ^5^Department of Epidemiology, School of Medicine, Jinan University, Guangzhou, China; ^6^Guangdong Key Laboratory of Environmental Exposure and Health, Jinan University, Guangzhou, China

**Keywords:** childhood neighborhood quality, cognitive function, body mass index, elders, Chinese

## Abstract

**Background**: Identification of early modifiable factors is crucial to delay or prevent the development of cognitive impairment and reduce the social and economic burden.

**Objective**: This study aimed to examine the longitudinal associations of childhood neighborhood quality (CNQ) with the risk of later-life cognitive dysfunction and the role of body mass index (BMI) in this association.

**Methods**: A total of 8,289 community-dwelling middle-aged and elderly population from wave 2011, wave 2013, and wave 2015 of the China Health and Retirement Longitudinal Study (CHARLS) were included. Cognitive function and CNQ were measured by standardized questionnaires. Multilevel linear regression models were used to estimate the associations of CNQ and cognitive function. The interactions of BMI with CNQ in the progress of cognitive function were also estimated.

**Results**: The participants with higher CNQ had a significantly low risk of cognitive impairment than those with lower CNQ score (*β* = 0.067, 95% CI: 0.031, 0.103), and the results remained similar (*β* = 0.039, 95% CI: 0.004, 0.075) after controlling other confounding variables. Furthermore, there was an interaction between BMI with CNQ score (*P* < 0.001) for the risk of cognitive impairment. In BMI-stratified analysis, we found that the association of CNQ and cognitive function was not statistically significant in overweight or obese population (*β* = 0.019, 95% CI: −0.032, 0.070), but was statistically significant in people with lower BMI (*β* = 0.059, 95% CI: 0.010, 0.107).

**Conclusions**: Higher CNQ score is significantly associated with the lower risk of cognitive dysfunction in adulthood. BMI may moderate the associations of CNQ with the risk of cognitive function.

## Introduction

Cognitive impairment is one of the most common health problems for elders worldwide (Jiang et al., 2020). In 2019, Alzheimer’s Disease International estimated that there are over 50 million people living with dementia globally, a figure set to increase to 152 million by 2050. The annual estimated worldwide cost of dementia is US$ 2 trillion, and this cost will be doubled by 2030 as the number of people with dementia continues to rise (Alzheimer’s Disease International, 2019). Therefore, identification of early modifiable factors is crucial to delay or prevent the development of cognitive impairment and reduce the social and economic burden.

Evidence demonstrated that living in a lower-quality neighborhood, such as with high crime rate and poverty, is associated with a higher elevated risk of physiological and psychological health (O’campo et al., 2015), including cognitive impairment. The data from the Hispanic Community Health Study/Study of Latinos (HCHS/SOL) and its Sociocultural Ancillary Study (SCAS) showed that middle-aged and older Hispanic/Latina women living in neighborhoods with the lowest perceived problems had higher global cognition and memory (Estrella et al., 2020). Brown et al. (2009) reported that a more positive neighborhood social environment was associated with better mental health outcomes in older Hispanic immigrants. The Cardiovascular Health Study showed that higher neighborhood socioeconomic status (SES) was associated with a greater general cognitive function at baseline but was not associated with differences in change of general cognitive function over 6 years (Rosso et al., 2016). The data from the Chicago Health and Aging Project also highlighted the role of neighborhood environments in buffering cognitive decline among older adults (Clarke et al., 2015).

Early life environments may play an important role in the development of cognitive impairment later in life, although previous studies indicated that neighborhood quality associated with cognitive function. However, the results were inconsistent, and most studies used a cross-sectional design or focused on older population (Brown et al., 2009; Murayama et al., 2013; Rosso et al., 2016; Estrella et al., 2020; Taylor et al., 2020). On the other hand, the association between body mass index (BMI) and cognitive dysfunction is far from clear (Coin et al., 2012; Delgado-Rico et al., 2012). Several studies reported that being overweight in mid-life was associated with the increased risk of cognitive dysfunction in later life (Kivipelto et al., 2005; Whitmer et al., 2008), while being overweight in late life (≥65 years) decreased the subsequent risk of dementia (Fitzpatrick et al., 2009). Moreover, compared with BMI in normal range, being underweight or having a decrease in BMI in late life was reported to increase the risk of dementia in elderly people (Fitzpatrick et al., 2009; Emmerzaal et al., 2015). One possible explanation might be the interactive effects of BMI and other risk factors on cognitive functioning. Building upon previous research, this study aimed to examine the longitudinal associations of childhood neighborhood quality (CNQ) with later-life cognitive function and the role of BMI in this association in a large representative Chinese population.

## Materials and Methods

### Study Population

The data were from the China Health and Retirement Longitudinal Study (CHARLS), which is a nationally representative longitudinal survey in China of the general population aged 45 years of age or older and their spouses (Zhao et al., 2014). The baseline survey was conducted between June 2011 and March 2012 and then followed every 2 years. The CHARLS respondents were sampled using a multistage probability sampling strategy and a probability proportional to size sampling technique. The detailed sampling design of the CHARLS is available elsewhere (Zhao et al., 2014). Briefly, the residents aged 45 years or older and their spouses living in China (including 28 provinces, municipal cities, and autonomous regions) were first conducted through face-to-face computer-assisted personal interviewing in June 2011, and these participants were followed every 2 years. The baseline survey was conducted with 17,708 individual participants, and the response rate among eligible households was 80.5%. In this study, the data of wave 2011, wave 2013, and wave 2015 were used. Among the study participants, 10,721 participants completed the cognitive function assessment at least at one wave; of them, 8289 individuals at the last wave with completed data on age, sex, BMI, education, marital status, smoking, drinking, exercise, family income, urban, chronic diseases, depressive symptoms, CNQ, and cognitive function were used in the current analyses. Supplementary Table 1 showed the differences between the participants who were included and those who are not included in the main analyses. CHARLS was approved by the Ethical Review Committee at Peking University, and all participants gave written informed consent before participation.

### Childhood Neighborhood Quality

CNQ was measured with four questions (Supplementary Table 2), for example, “Was it safe being out alone at night in the neighborhood where you lived as a child? (Not safe at all = 0, Not very safe = 1, Somewhat safe = 2, Very safe = 3).” The total CNQ score of the scale ranged from 0 to 12, with a higher score indicating the higher level of neighborhood quality (Chen et al., 2020).

### Cognitive Function

The cognitive function was assessed using the following three categories: (1) Episodic memory. The respondents were asked to repeat as many words as they could recall from a list of 10 simple Chinese nouns read to him/her (immediate word recall) and to repeat the same after 5 min (delayed recall; Wang et al., 2017). The episodic memory score, ranging from 0 to 10, was calculated by the average correct number of immediate and delayed word recalls; (2) Telephone Interview of Cognitive Status (TICS). Ten mental status items were selected from TICS to measure the orientation and attention. These questions included the awareness of the date (year, month, and day), the day of the week, season of the year, and a Serial 7’s subtraction test beginning with 100 and ending at 65. Also, all participants were asked if the paper and pencil or other aid was needed when completing the number subtraction (NO = 1 and YES = 0). The score in this category ranged from 0 to 11; and (3) Figure drawing. Respondents were shown a picture of two overlapped pentagons and asked to draw a similar figure (Huang and Zhou, 2013). Respondents who successfully completed the task received a score of 1, and those who failed received a score of 0. The overall cognition score was calculated by these three dimensions, ranging from 0 to 22. The higher score indicated the better cognitive function.

### Health and Lifestyle Measurements

Weight and height were measured by trained technicians who used standardized procedures. BMI was calculated as weight in kilograms divided by the square of height in meters. Lifestyle and personal health-related behaviors were collected using a standardized questionnaire. Participants taking moderate/vigorous exercise at least 10 min/day and at least 3 days during a usual week were defined as exercise. Current smokers were defined as smoking at least 1 cigarette per day and currently smoking. Participants were categorized as never drinkers (drink any alcoholic beverages more than once a month in the past years), former drinkers (used to drink any alcoholic beverages more than once a month in the past), and non-drinkers. High education was defined as having at least 12 years of education (high school or above). The question “When you were a child before age 17, compared to the average family in the same community/village at that time, how was your family’s financial situation?” formed the basis for categories of family’s financial situation (better off, same as, and worse off). Depressive symptom score was measured by the 10-item Center for Epidemiological Studies—Depression Scale (CES—D10; Zhao et al., 2014).

### Statistical Methods

Continuous variables are presented as mean ± SD, whereas categorical variables are presented as cases (*n*) and percentage (%). Differences were examined by the two-sample *t*-test for continuous variables or by the chi-square test for categorical variables. Multilevel linear regression models (community–household–individual) were used to estimate the associations of CNQ with cognitive function. The data structure was that individual values were clustered within households, which in turn were clustered within the community. Univariate analyses were performed in Model 1. Model 2 was adjusted for age and sex. In Model 3, categorical variables (education, marital status, smoking, drinking, exercise, family income, and urban) and continuous variables (BMI and depressive symptoms) were further controlled. In addition, the interactions of BMI with CNQ in the risk of cognitive function was also estimated, in which BMI was treated as a categorical variable (≥24 and <24 kg/m^2^). Considering the data were missing not at random (Supplementary Table 1), we performed a sensitivity analysis further introducing the repeated measurements of cognitive function in the model (community–household–individual–repeated measurement). All analyses were performed using Stata software version 14 (STATA Corporation, TX, US). A two-sided *P* < 0.05 was considered statistically significant.

## Results

The characteristics of the participants are presented in Table 1. Overall, the mean age was 56.8 ± 9.3 years, and 3875 (46.8%) were female. The score of CNQ was 8.9 ± 1.7, and the cognitive function score was 14.7 ± 3.0. The characteristics were statistically significant differences between males and females except for exercise and living in rural area (*P* > 0.05).

**Table 1:** Characteristics of participants.

	Males (*n* = 3,875)	Females (*n* = 4,414)	*P*-value
Age (years)	58.1 (57.9–58.4)	55.4 (55.1–55.7)	<0.001
Body mass index (kg/m^2^)	23.7 (23.6–23.8)	24.6 (24.4–24.7)	<0.001
High school education or higher (%)	54.6 (53.1–56.1)	50.3 (48.8–51.9)	<0.001
Currently married (%)	93.0 (92.2–93.7)	90.2 (89.2–91.1)	<0.001
Smoking (%)	54.3 (52.8–55.7)	4.9 (4.2–5.6)	<0.001
Drinking (%)			
None	40.3 (38.8–41.7)	90.1 (89.2–91)	<0.001
Current	11.5 (10.6–12.5)	2.5 (2.1–3.1)	
Former	48.2 (46.8–49.7)	7.4 (6.6–8.2)	
Exercise (%)	29.7 (28.3–31)	29.5 (28.1–30.9)	0.895
Family’s financial situation (%)			
Better off	8.7 (7.9–9.6)	12.5 (11.5–13.6)	<0.001
Same as	54.4 (53.0–55.9)	55.6 (54.0–57.1)	
Worse off	36.8 (35.5–38.3)	31.9 (30.5–33.4)	
Living in rural area (%)	82.8 (81.6–83.8)	82.0 (80.8–83.2)	0.373
Depression symptom score*	8.6 (8.5–8.7)	9.8 (9.7–10.0)	<0.001
Childhood neighborhood quality score	8.8 (8.8–8.9)	9.0 (9.0–9.1)	<0.001
Cognitive function score	14.8 (14.7–14.9)	14.5 (14.4–14.6)	<0.001

*Measured by the 10-item Center for Epidemiological Studies–Depression Scale.

**Figure 1:**
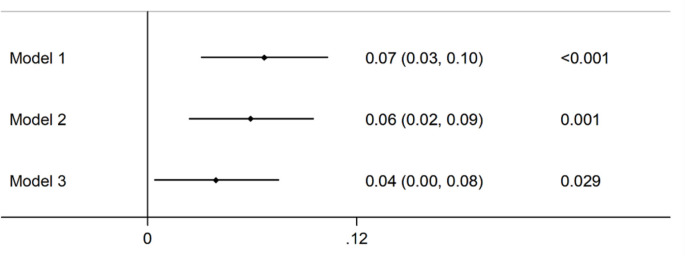
Association of childhood neighborhood quality (CNQ) and cognitive function in adults. Model 1: unadjusted; Model 2: adjusted for age, sex; Model 3: adjusted for age, sex, body mass index (BMI), education, marital status, smoking, drinking, exercise, family income, urban, and depressive symptoms.

The association between CNQ and cognitive function in late adulthood is shown in Figure 1. The participants with higher CNQ had a significant low risk of cognitive impairment than those with lower CNQ score (*β* = 0.067, 95% CI: 0.031, 0.103;* P* < 0.001), and further adjusted for age, sex, BMI, education, marital status, smoking, drinking, exercise, family income, urban, and depressive symptoms, the results remained similar (*β* = 0.039, 95% CI: 0.004, 0.075;* P* = 0.001).

**Figure 2:**
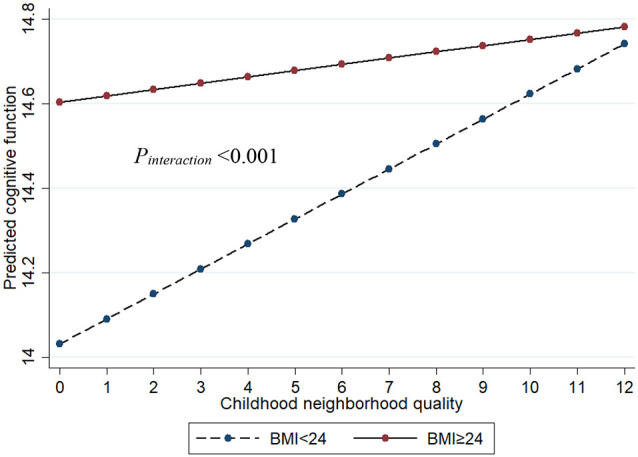
The interaction between BMI and CNQ on cognitive function. Age, sex, education, marital status, smoking, drinking, exercise, family income, urban, and depressive symptoms were adjusted in the model.

There was an interaction between BMI with CNQ score (*P*_interaction_ < 0.001) for the risk of cognitive impairment, which suggests that BMI moderates the associations between CNQ and the risk of cognitive impairment (Figure 2). In BMI-stratified analysis, we found that the association of CNQ and cognitive function was not statistically significant in overweight or obese population (*β* = 0.019, 95% CI: −0.032, 0.070;* P* = 0.467); however, one CNQ score increase was associated with a 0.059 increase of cognitive function score in a population with lower BMI (*β* = 0.059, 95% CI: 0.010, 0.107; *P* = 0.018; Table 2).

**Table 2:** Association of childhood neighborhood quality and cognitive function in adults by body mass index.

	Body mass index ≥24 (*N* = 3,939)			Body mass index <24 (*N* = 4,350)		
	*β*	95% CI	*P*-value	*β*	95% CI	*P*-value
Model 1	0.045	−0.007, 0.098	0.091	0.092	0.042, 0.141	<0.001
Model 2	0.049	−0.002, 0.100	0.062	0.078	0.030, 0.127	0.002
Model 3	0.019	−0.032, 0.070	0.467	0.059	0.010, 0.107	0.018

*CI, confidence interval. Model 1: unadjusted; Model 2: adjusted for age, sex; Model 3: adjusted for age, sex, body mass index, education, marital status, smoking, drinking, exercise, family income, urban, and depressive symptoms*.

Sensitivity analyses further introduced the repeated measurements of cognitive function in the multilevel model, and a total of 10,721 participants with 13,843 measurements were included in the analyses. Similar results were found and presented in Supplementary Table 3.

## Discussion

We found that higher CNQ was significantly associated with the lower risk of cognitive function in adults, and the association was independent of SES, unhealthy behaviors, and depressive symptoms of the guardian. To our best knowledge, we firstly reported that there was an interaction of CNQ score with BMI level for the risk of cognitive dysfunction, indicating that high BMI moderated the associations between CNQ and the risk of cognitive dysfunction.

A growing body of literature has reported that the neighborhood environments could predict the cognitive functions across the lifespan (Leventhal and Brooks-Gunn, 2000; Sharkey and Elwert, 2011; Fernandez-Blazquez et al., 2020). Richard et al. found that older adults living with low neighborhood-level educational attainment achieved lower cognitive function after controlling individual-level education and contextual-level median household income (Wight et al., 2006). Another research including 2,802 community-dwelling older adults (aged 65–94 years) showed that neighborhood SES position independently predicted late-life vocabulary function, but not in general cognition, processing speed, reasoning, and everyday cognition (Sisco and Marsiske, 2012). One similar study investigated by Brandt found the associations between SES and composite cognitive measures of cognitive status, vocabulary, and verbal short memory in children.

Another essential CNQ indexes is neighborhood deprivation, which was defined as a critical environmental vulnerability factor lacking resources (e.g., scarcity of educational and economic resources) necessary for healthy development (Cubbin and Winkleby, 2005; Mclaughlin et al., 2014). It has been reported that neighborhood deprivation was adversely associated with neurodevelopment and cognitive function (Krishnadas et al., 2013; Mclaughlin et al., 2014). For example, the Adolescent Brain Cognitive Development (ABCD) study with 10,205 youth showed that the neighborhood deprivation predicted lower cognitive function including lower neurocognitive performance and distinct prefrontal gray matter features after controlling the variables of parental education and household income (Vargas et al., 2020). Another study conducted in the United Kingdom also revealed a clear and significant downward trend of cognitive function score and testing of verbal fluency and memory for older adults with greater neighborhood deprivation exposure (Lang et al., 2008).

Underlying mechanisms between CNQ and cognitive function have not been fully studied. One potential explanation could be biosocial ecological theory, which refers to the interactions between individual’s development and their childhood environment (Bronfenbrenner and Morris, 1998). Children growing up in neighborhood with poor quality may have less access to positive social conditions, such as social cohesions and parent–child interaction (Nettle and Cockerill, 2010; Hanson et al., 2011), which may lead the poor cognitive function in the future. It has been reported that, compared to children from high-income neighborhoods, those from low-income communities have lower cognitive assessment scores, poorer health behaviors (e.g., help-seeking), and more chances of food insecurity (Kimbro and Denney, 2013; Brotman et al., 2016; Morrissey et al., 2016). Several studies further demonstrated that the environment of the childhood neighborhood was one of the determinants of their short- and long-term physical and mental health patterns, academic skills, and economic status in the future (Brotman et al., 2016; Chetty et al., 2016; Wolf et al., 2017).

Numerous studies have investigated the linkage between BMI and cognitive dysfunction or dementia (Coin et al., 2012; Delgado-Rico et al., 2012), but the results are very mixed. Some studies showed that higher baseline BMI was associated with increased risk of dementia and cognitive function (Gustafson et al., 2012; Gu et al., 2014; Deckers et al., 2017), while others observed an inverse association in individuals at age 65 years and older (Hughes et al., 2009; Nettle and Cockerill, 2010; Tolppanen et al., 2014). Some studies even reported a U-shaped relation between BMI and dementia at older ages (Luchsinger et al., 2007; Beydoun et al., 2008; Michaud et al., 2018b). Our results were in line with previous studies in which the authors reported that high baseline BMI was associated with slower progression of functional or cognitive declines (Besser et al., 2014; Michaud et al., 2018a). This study showed that there is an interaction between CNQ score with BMI value for the risk of cognitive dysfunction, suggesting that CNQ may mutually modify the association between BMI and cognitive function. This may be a novel avenue to explain the “obesity paradox,” and it should be considered in the future studies.

### Limitations and Strengths

One major strength was that we investigated the longitudinal associations between CNQ score with cognitive function using a large, nationally representative sample. Another strength point was that we adjusted as much confounding as possible in our study, such as childhood SES and depressive symptoms in the male and female guardian, etc., which might be associated with cognitive function in adults. There were also several limitations in our study. One limitation is that not all risk factors for cognition assessment, such as genotypes and trauma, were available in this study. Another limitation is that CNQ data were retrospectively assessed, and self-report measures of CNQ data may be sensitive to recall bias.

## Conclusion

In conclusion, this study showed that higher CNQ was significantly associated with the lower score of cognitive assessment in adults. For the first time, we reported that high BMI moderates the association between CNQ and the risk of cognitive dysfunction, indicating that considering interactions of BMI and other risk factors of cognitive dysfunction in further studies may help to explain the “obesity paradox.” Establishing a comprehensive community, family, and government intervention model to improve neighborhood quality may reduce the burden of cognitive dysfunction in the population. Also, the interactions between BMI and other covariances should be considered in further studies exploring the risk factors of cognitive dysfunction.

## Data Availability Statement

Publicly available datasets were analyzed in this study. This data can be found here: the data of this study are openly available at http://charls.pku.edu.cn/index/en.html.

## Ethics Statement

The studies involving human participants were reviewed and approved by Ethical Review Committee at Peking University. The patients/participants provided their written informed consent to participate in this study.

## Author Contributions

GH and CJ: had full access to all of the data in the study and take responsibility for the integrity of the data and the accuracy of the data analysis. GH and CJ: concept and design. PX and XL: acquisition, analysis, or interpretation of data, and statistical analysis. PX: drafting of the manuscript. XL, HC, LC, LZ, CJ, and GH: critical revision of the manuscript for important intellectual content, administrative, technical, or material support. CJ and GH: supervision. All authors contributed to the article and approved the submitted version.

## Conflict of Interest

The authors declare that the research was conducted in the absence of any commercial or financial relationships that could be construed as a potential conflict of interest.

## References

[B1] Alzheimer’s Disease International. (2019). Alzheimer’s Disease International. World Alzheimer Report 2019: Attitudes to Dementia. Available online at: https://www.alzint.org/resource/world-alzheimer-report-2019/.

[B2] Besser L. M. Gill D. P. Monsell S. E. Brenowitz W. Meranus D. H. Kukull W.. (2014). Body mass index, weight change and clinical progression in mild cognitive impairment and Alzheimer disease. Alzheimer Dis. Assoc. Disord. 28, 36–43. 10.1097/WAD.0000000000000005 24126214 PMC3945175

[B3] Beydoun M. A. Beydoun H. A. Wang Y. (2008). Obesity and central obesity as risk factors for incident dementia and its subtypes: a systematic review and meta-analysis. Obes. Rev. 9, 204–218. 10.1111/j.1467-789X.2008.00473.x 18331422 PMC4887143

[B4] Bronfenbrenner U. Morris P. A. (1998). The ecology of developmental processes. Handbook Child Psychol. 1, 993–1028.

[B5] Brotman L. M. Dawson-Mcclure S. Kamboukos D. Huang K. Y. Calzada E. J. Goldfeld K.. (2016). Effects of parentcorps in prekindergarten on child mental health and academic performance follow-up of a randomized clinical trial through 8 years of age. JAMA Pediatr. 170, 1149–1155. 10.1001/jamapediatrics.2016.1891 27695851 PMC5642293

[B6] Brown S. C. Mason C. A. Spokane A. R. Cruza-Guet M. C. Lopez B. Szapocznik J. (2009). The relationship of neighborhood climate to perceived social support and mental health in older hispanic immigrants in miami, florida. J. Aging Health 21, 431–459. 10.1177/0898264308328976 19318605 PMC2933404

[B7] Chen H. Y. Xiong P. Chen L. Hao G. (2020). Childhood neighborhood quality, friendship and risk of depressive symptoms in adults: the China health and retirement longitudinal study. J. Affect. Disord. 276, 732–737. 10.1016/j.jad.2020.07.090 32736183

[B8] Chetty R. Hendren N. Katz L. F. (2016). The effects of exposure to better neighborhoods on children: new evidence from the moving to opportunity experiment. Am. Econ. Rev. 106, 855–902. 10.1257/aer.20150572 29546974

[B9] Clarke P. J. Weuve J. Barnes L. Evans D. A. De Leon C. F. M. (2015). Cognitive decline and the neighborhood environment. Ann. Epidemiol. 25, 849–854. 10.1016/j.annepidem.2015.07.001 26253697 PMC4609590

[B10] CoinA.VeroneseN.De RuiM.MoseleM.BolzettaF.GirardiA.. (2012). Nutritional predictors of cognitive impairment severity in demented elderly patients: the key role of BMI. J. Nutr. Health Aging 16, 553–556. 10.1007/s12603-012-0052-x22659996

[B11] Cubbin C. Winkleby M. A. (2005). Protective and harmful effects of neighborhood-level deprivation on individual-level health knowledge, behavior changes and risk of coronary heart disease. Am. J. Epidemiol. 162, 559–568. 10.1093/aje/kwi250 16093286

[B12] DeckersK.Van BoxtelM. P. J.VerheyF. R. J.KohlerS. (2017). Obesity and cognitive decline in adults: effect of methodological choices and confounding by age in a longitudinal study. J. Nutr. Health Aging 21, 546–553. 10.1007/s12603-016-0757-328448085

[B13] Delgado-Rico E. Rio-Valle J. S. Gonzalez-Jimenez E. Campoy C. Verdejo-Garcia A. (2012). BMI predicts emotion-driven impulsivity and cognitive inflexibility in adolescents with excess weight. Obesity 20, 1604–1610. 10.1038/oby.2012.47 22421897

[B14] Emmerzaal T. L. Kiliaan A. J. Gustafson D. R. (2015). 2003–2013: a decade of body mass index, Alzheimer’s disease and dementia. J. Alzheimers Dis. 43, 739–755. 10.3233/JAD-141086 25147111

[B15] Estrella M. L. Durazo-Arvizu R. A. Gallo L. C. Isasi C. R. Perreira K. M. Vu T. H. T.. (2020). Associations between perceived neighborhood environment and cognitive function among middle-aged and older women and men: hispanic community health study/study of latinos sociocultural ancillary study. Soc. Psychiatry Psychiatr. Epidemiol. 55, 685–696. 10.1007/s00127-019-01829-0 31974810 PMC7276286

[B16] Fernandez-Blazquez M. A. Noriega-Ruiz B. Avila-Villanueva M. Valenti-Soler M. Frades-Payo B. Del Ser T.. (2020). Impact of individual and neighborhood dimensions of socioeconomic status on the prevalence of mild cognitive impairment over seven-year follow-up. Aging Ment. Health, 1–10. [Online ahead of print]. 10.1080/13607863.2020.1725803 32067489

[B17] Fitzpatrick A. L. Kuller L. H. Lopez O. L. Diehr P. O’meara E. S. Longstreth W. T.. (2009). Midlife and late-life obesity and the risk of dementia: cardiovascular health study. Arch. Neurol. 66, 336–342. 10.1001/archneurol.2008.582 19273752 PMC3513375

[B18] Gu Y. Scarmeas N. Cosentino S. Brandt J. Albert M. Blacker D.. (2014). Change in body mass index before and after Alzheimer’s disease onset. Curr. Alzheimer Res. 11, 349–356. 10.2174/1567205010666131120110930 24251397 PMC4026350

[B19] Gustafson D. R. Backman K. Joas E. Waern M. Ostling S. Guo X. X.. (2012). 37 years of body mass index and dementia: observations from the prospective population study of women in Gothenburg, Sweden. J. Alzheimers Dis. 28, 162–171. 10.3233/JAD-150326 21965312

[B20] Hanson M. J. Miller A. D. Diamond K. Odom S. Lieber J. Butera G.. (2011). Neighborhood community risk influences on preschool children’s development and school readiness. Infants Young Child. 24, 87–100. 10.1097/IYC.0b013e3182008dd0

[B21] Huang W. Zhou Y. (2013). Effects of education on cognition at older ages: evidence from China’s great famine. Soc. Sci. Med. 98, 54–62. 10.1016/j.socscimed.2013.08.021 24331882

[B22] Hughes T. F. Borenstein A. R. Schofield E. Wu Y. Larson E. B. (2009). Association between late-life body mass index and dementia: the Kame Project. Neurology 72, 1741–1746. 10.1212/WNL.0b013e3181a60a58 19451529 PMC2683740

[B23] Jiang N. Wu B. Lu N. Dong T. (2020). Neighborhood-based social capital and cognitive function among older adults in five low- and middle-income countries: evidence from the world health organization study on global AGEing and adult health. Int. J. Geriatr. Psychiatry 35, 365–375. 10.1002/gps.5239 31755134

[B24] Kimbro R. T. Denney J. T. (2013). Neighborhood context and racial/ethnic differences in young children’s obesity: structural barriers to interventions. Soc. Sci. Med. 95, 97–105. 10.1016/j.socscimed.2012.09.032 23089614

[B25] Kivipelto M. Ngandu T. Fratiglioni L. Viitanen M. Kåreholt I. Winblad B.. (2005). Obesity and vascular risk factors at midlife and the risk of dementia and Alzheimer disease. Arch. Neurol. 62, 1556–1560. 10.1001/archneur.62.10.1556 16216938

[B26] Krishnadas R. Mclean J. Batty G. D. Burns H. Deans K. A. Ford I.. (2013). Socioeconomic deprivation and cortical morphology: psychological, social and biological determinants of ill health study. Psychosom. Med. 75, 616–623. 10.1097/PSY.0b013e3182a151a7 23975946

[B27] Lang I. A. Llewellyn D. J. Langa K. M. Wallace R. B. Huppert F. A. Melzer D. (2008). Neighborhood deprivation, individual socioeconomic status and cognitive function in older people: analyses from the English longitudinal study of ageing. J. Am. Geriatr. Soc. 56, 191–198. 10.1111/j.1532-5415.2007.01557.x 18179489 PMC2671806

[B28] Leventhal T. Brooks-Gunn J. (2000). The neighborhoods they live in: the effects of neighborhood residence on child and adolescent outcomes. Psychol. Bull. 126, 309–337. 10.1037/0033-2909.126.2.309 10748645

[B29] Luchsinger J. A. Patel B. Tang M. X. Schupf N. Mayeux R. (2007). Measures of adiposity and dementia risk in elderly persons. Arch. Neurol. 64, 392–398. 10.1001/archneur.64.3.392 17353383 PMC1821350

[B30] Mclaughlin K. A. Sheridan M. A. Lambert H. K. (2014). Childhood adversity and neural development: deprivation and threat as distinct dimensions of early experience. Neurosci. Biobehav. Rev. 47, 578–591. 10.1016/j.neubiorev.2014.10.012 25454359 PMC4308474

[B31] Michaud T. L. Siahpush M. Farazi P. A. Kim J. Yu F. Su D.. (2018a). The association between body mass index and cognitive, functional and behavioral declines for incident dementia. J. Alzheimers Dis. 66, 1507–1517. 10.3233/JAD-180278 30412484 PMC6441968

[B32] Michaud T. L. Siahpush M. Farazi P. A. Kim J. Yu F. Su D. J.. (2018b). The association between body mass index and cognitive, functional and behavioral declines for incident dementia. J. Alzheimers Dis. 66, 1507–1517. 10.3233/JAD-180278 30412484 PMC6441968

[B33] Morrissey T. W. Oellerich D. Meade E. Simms J. Stock A. (2016). Neighborhood poverty and children’s food insecurity. Child. Youth Serv. Rev. 66, 85–93. 10.1016/j.childyouth.2016.05.006

[B34] Murayama H. Nishi M. Matsuo E. Nofuji Y. Shimizu Y. Taniguchi Y.. (2013). Do bonding and bridging social capital affect self-rated health, depressive mood and cognitive decline in older Japanese? A prospective cohort study. Soc. Sci. Med. 98, 247–252. 10.1016/j.socscimed.2013.09.026 24331905

[B35] Nettle D. Cockerill M. (2010). Development of social variation in reproductive schedules: a study from an english urban area. PLoS One 5:e12690. 10.1371/journal.pone.0012690 20856795 PMC2939869

[B36] O’campo P. Wheaton B. Nisenbaum R. Glazier R. H. Dunn J. R. Chambers C. (2015). The neighbourhood effects on health and well-being (NEHW) study. Health Place 31, 65–74. 10.1016/j.healthplace.2014.11.001 25463919

[B37] Rosso A. L. Flatt J. D. Carlson M. C. Lovasi G. S. Rosano C. Brown A. F.. (2016). Neighborhood socioeconomic status and cognitive function in late life. Am. J. Epidemiol. 183, 1088–1097. 10.1093/aje/kwv337 27257114 PMC4908209

[B38] Sharkey P. Elwert F. (2011). The legacy of disadvantage: multigenerational neighborhood effects on cognitive ability. Am. J. Sociol. 116, 1934–1981. 10.1086/660009 21932471 PMC3286027

[B39] Sisco S. M. Marsiske M. (2012). Neighborhood influences on late life cognition in the ACTIVE study. J. Aging Res. 2012: 435826. 10.1155/2012/435826 22966458 PMC3433144

[B40] Taylor R. L. Cooper S. R. Jackson J. J. Barch D. M. (2020). Assessment of neighborhood poverty, cognitive function and prefrontal and hippocampal volumes in children. JAMA Netw. Open 3:e2023774. 10.1001/jamanetworkopen.2020.23774 33141160 PMC7610187

[B41] Tolppanen A. M. Ngandu T. Kareholt I. Laatikainen T. Rusanen M. Soininen H.. (2014). Midlife and late-life body mass index and late-life dementia: results from a prospective population-based cohort. J. Alzheimers Dis. 38, 201–209. 10.3233/JAD-130698 23948937

[B42] Vargas T. Damme K. S. F. Mittal V. A. (2020). Neighborhood deprivation, prefrontal morphology and neurocognition in late childhood to early adolescence. Neuroimage 220:117086. 10.1016/j.neuroimage.2020.117086 32593800 PMC7572635

[B43] Wang T. Wu Y. L. Sun Y. Y. Zhai L. Zhang D. F. (2017). A prospective study on the association between uric acid and cognitive function among middle-aged and older chinese. J. Alzheimers Dis 58, 79–86. 10.3233/JAD-161243 28387669

[B44] Whitmer R. A. Gustafson D. R. Barrett-Connor E. Haan M. N. Gunderson E. P. Yaffe K. (2008). Central obesity and increased risk of dementia more than three decades later. Neurology 71, 1057–1064. 10.1212/01.wnl.0000306313.89165.ef 18367704

[B45] Wight R. G. Aneshensel C. S. Miller-Martinez D. Botticello A. L. Cummings J. R. Karlamangla A. S.. (2006). Urban neighborhood context, educational attainment and cognitive function among older adults. Am. J. Epidemiol. 163, 1071–1078. 10.1093/aje/kwj176 16707655

[B46] Wolf S. Magnuson K. A. Kimbro R. T. (2017). Family poverty and neighborhood poverty: links with children’s school readiness before and after the great recession. Child. Youth Serv. Rev. 79, 368–384. 10.1016/j.childyouth.2017.06.040 30147212 PMC6107082

[B47] Zhao Y. H. Hu Y. S. Smith J. P. Strauss J. Yang G. H. (2014). Cohort profile: the china health and retirement longitudinal study (CHARLS). Int. J. Epidemiol. 43, 61–68. 10.1093/ije/dys203 23243115 PMC3937970

