# Supplementary material for: Association Between Childhood Neighborhood Quality and the Risk of Cognitive Dysfunction in Chinese Middle-Aged and Elderly Population: The Moderation Effect of Body Mass Index
Source: Front Aging Neurosci. 2021 May 13;13:645189. doi: 10.3389/fnagi.2021.645189 (PMC8155274; doi:10.3389/fnagi.2021.645189)
Supplement: Supplementary file 1 [file Table_1.docx]

Supplementary materials

| STable 1. Participants who were included and those were excluded from the analyses | | | |
| --- | --- | --- | --- |
| Characteristics | Included | Excluded | *P* value |
| Age (years) | 57.1(56.9-57.2) | 57.0(56.6-57.4) | 0.780 |
| Gender (women) | 53.3(52.2-54.4) | 56.1(54.2-58.1) | 0.013 |
| Rural (%) | 81.8(81.0-82.0) | 74.9(73.1-76.5) | <0.001 |

| STable 2. Questions of childhood neighborhood quality | |
| --- | --- |
| Q1. Was it safe being out alone at night in the neighborhood where you lived as a child? Is it very safe, somewhat safe, not very safe or not safe at all? | Not safe at all =0, Not very safe =1, Somewhat safe =2, Very safe =3 |
| Q2. Were the neighbors of the place where you lived as a child willing to help each other out? Is it very willing to, somewhat willing to, not very willing to or not willing to? | Not willing to at all =0, Not very willing to =1, Somewhat willing to =2, Very willing to =3 |
| Q3. Were the neighbors of the place where you lived as a child very close-knit? Is it very close-knit, somewhat close-knit, not very close-knit or not close-knit at all? | Not close-knit at all =0, Not very close-knit =1, Somewhat close-knit =2, Very close-knit =3 |
| Q4. Was the neighborhood of the place where you lived as a child very clean and attractive? Is it very clean and attractive, somewhat clean and attractive, not very clean and attractive or not clean and attractive at all? | Not clean and attractive at all =0, Not very clean and attractive=1, Somewhat clean and attractive=2, Very clean and attractive =3 |

| STable 3. Association of childhood neighborhood quality and cognitive function in adults (Sensitivity analysis) | | | |
| --- | --- | --- | --- |
|  | β | 95%CI | *P* value |
| Model 1 | 0.064 | 0.035, 0.092 | <0.001 |
| Model 2 | 0.051 | 0.023, 0.079 | <0.001 |
| Model 3 | 0.034 | 0.004, 0.064 | 0.025 |
| CI = confidence interval  Model 1: unadjusted; Model 2: adjusted for age, sex; Mode 3: adjusted for age, sex, body mass index, education, marital status, smoking, drinking, exercise, family income, urban, and depressive symptoms  A total of 10,721 participants with 13,843 measurements were included in the model | | | |
